# Ab Initio Study on Dopant Relaxation Mechanism in Ti and Ce Cationically Substituted in Wurtzite Gallium Nitride

**DOI:** 10.3390/ma15103599

**Published:** 2022-05-18

**Authors:** Mohammad Alkhedher, Abdul Majid, Niyazi Bulut, Samah Elsayed Elkhatib

**Affiliations:** 1 Mechanical and Industrial Engineering Department, Abu Dhabi University, Abu Dhabi 111188, United Arab Emirates; mohammad.alkhedher@adu.ac.ae; 2 Department of Physics, University of Gujrat, Gujrat 50700, Pakistan; 3 Department of Physics, Faculty of Science, Firat University, Elaziğ 23119, Turkey; nbulut@firat.edu.tr; 4 Mechanical Engineering Department, Faculty of Engineering and Technology, Future University in Egypt, New Cairo 11835, Egypt; samah.elmetwally@fue.edu.eg

**Keywords:** relaxation, cationic sites, doping, density functional theory

## Abstract

The changes in properties of materials upon introduction of impurities is well documented but less is known about the location of foreign atoms in different hosts. This study is carried out with the motivation to explore dopant location in hexagonal GaN using density functional theory based calculations. The dopant site location of the individual dopants Ti, Ce, and Ti-Ce codoped wurtzite GaN was investigated by placing the dopants at cationic lattice sites as well as off-cationic sites along the c-axis. The geometry optimization relaxed individual dopants on cationic Ga sites but in the case of codoping Ce settled at site 7.8% away along $\left[000\bar{1\;}\right]$ and Ti adjusted itself at site 14% away along [0001] from regular cationic sites. The analysis of the results indicates that optimized geometry is sensitive to the starting position of the dopants. The magnetic exchange interactions between Ti and Ce ions are responsible for their structural relaxation in the matrix.

## 1. Introduction

The introduction of impurity atoms into semiconducting hosts via doping is essentially required for device grade as it helps to tailor the material properties via defect engineering [1,2]. The doping is considered primarily as the substitutional placement of foreign atoms on lattice sites of the matrix to ensure the activation of the dopants while maintaining the original structure of the host material. The substitutional incorporation of the dopant ions is capable of changing electronic structure, catalytic activity, light absorption or transmission, luminescence, and introducing magnetic properties to the materials [3]. Doping of foreign atoms into elemental and compound hosts is carried out either during synthesis or after growth via different methods [4]. 

The determination of the dopant’s location in the host material is always challenging and requires careful characterizations for the purpose [5]. The experimentalists make special arrangements including thermal annealing to activate the dopants incorporated in situ or via ion-implantation [6]. The experimental conditions are optimized by considering the detection of dopant-related electrical, optical or magnetic signatures. However, the situation is complicated when modeling of the process is theoretically performed. The modeling sometimes produces superficial effects due to several mechanisms which are often neglected, e.g., homogeneous doping, dopant to host atoms ratio, alloying, limited solubility due to atomic size differences, thermodynamics of doping, deep-centers that remain unionized at STP, compensation to negate the doping, calculations at 0° K, insufficient geometry optimization, supercell effects, dopant location, etc. In order to computationally model the process, the placement of the dopants at lattice sites is usually simulated via first-principles methods. The rich literature in this regard points to the strategy of inserting the foreign atoms in the host followed by geometry optimization which usually relaxes the structure to settle the dopant either precisely on cationic lattice sites or in proximate locations [3,4,5,6]. The properties of the doped materials strongly depend upon the dopant’s location in the host due to which placement of the dopants should be carefully handled [7]. The experimental determination of dopant site location is carried out using different techniques including X-ray diffraction, extended X-ray absorption fine structure spectroscopy, X-ray photoelectron spectroscopy, Rutherford Backscattering Spectroscopy, electron channeling techniques, etc. [1,7,8]. However, not only the experimental studies are usually reported without confirming the dopant’s location but also the computational studies merely consider the true location of the impurity atoms in the host matrix. It is thus producing disagreements in findings and makes the dopant-related research less useful for the realization of the materials into applications and devices.

The precise determination of dopant location is necessary to accurately predict the properties of doped materials [9]. The literature exhibits contradictory reports on the same dopant–host systems which are either due to differences in experimental conditions, computational recipes, or dopant location issues. The doping of Cr^3+^ is reported to exhibit contradictory results in the form of an increase as well as a decrease in photoactivity of host TiO_2_ [1,10,11]. Prior to geometry optimization, the dopant may be inserted in two ways; first by placing on a host lattice site, and secondly insertion off-site with a vacancy on the target site. The structural relaxation in the first case usually settles the dopant close to the lattice site whereas in the second case the dopant may be seated at a location in the path during diffusion to the vacancy [12]. The relaxation of the dopant precisely at a host lattice site is not guaranteed but this is the most assumed situation in reported literature [8]. The second situation is closer to the real doping process where a driving force is present to diffuse the dopant atoms to find a suitable equilibrium location [8]. In the authors’ opinion, lack of knowledge of the dopant’s location in host materials is one of the reasons for disagreement in the reported results and lack of utilization of doped materials in device grade applications. 

This study is carried out with the objective to investigate the mechanism of Ti and Ce dopants’ location in GaN using first-principles calculations. Gallium Nitride (GaN) is such a wide and direct bandgap semiconducting material which offers attractive chemical and physical properties for electronic and optoelectronic applications. It is considered a good host for accepting heavy doping of rare earth and transition metal atoms at high solubility to realize high Curie temperature diluted magnetic semiconductors. The environment of GaN is suitable for doping because it is known to activate the electrical, optical, and magnetic character of the dopants with low thermal quenching. The dopant Ti: [Ar] 3d² 4s² will be in form of Ti^3+^: 3d^1^ and Ce: [Xe] 4f¹ 5d¹ 6s² will be in Ce^3+^:4f^1^ charge state, considering triply ionized forms, when doped in GaN. The simple valance electronic configuration with one valance electron related to the dopants will be helpful to avoid complications arising from spin-pairing and exchange interactions. These dopants are considered suitable to study dopant relaxation mechanisms in wide gap semiconductors such as GaN.

## 2. Computational Details

Density functional theory based calculations were performed using the plane wave technique implemented in Vienna Ab initio Simulation Package (VASP). The calculations were performed at GGA-PBE and GGA + U levels of theory. The potentials for atoms were selected under frozen core scheme thereby considering valance configurations as Ga [4s^2^3d^10^4p^1^], N [2s^2^2p^3^], Ti [4s^2^3d^2^] and Ce [4f^1^5d^1^6s^2^]. Therefore, plane wave basis set was employed for the mentioned valance electrons whereas pseudopotentials were utilized to handle the core electrons. The relaxed configuration of wurtzite GaN was employed by adopting experimental lattice constants of a = 3.189 Å and c = 5.185 Å and internal parameter 0.377 as starting parameters [13]. The calculations were carried out using cut off energy 460 eV that appeared to sufficiently compute wave functions and charge density. The structures were relaxed with energy convergence of 10^−6^ and gradients criteria 0.001 eV/Å. The conjugate gradient algorithm was used for ionic relaxation whereas the stress tensor was computed with complete relaxations in cell shape and cell volume. The integration over the first Brillouin zone was conducted using the optimized value of Monkhorst–Pack k-point mesh 5 × 5 × 5. For good convergence and precision sampling for BZ integration, we used the tetrahedron smearing method with Blöchl corrections. In order to check the supercell size effects and find the numerical error, we created artificial units in form of supercells of different sizes including 1 × 1 × 1, 2 × 2 × 2, 3 × 3 × 2, 4 × 4 × 2, 4 × 4 × 4, 5 × 5 × 3 were used. The value of numerical error for the heat of formation, length, and angle is found to be ±0.002 eV, ±0.001 Å, and ±0.01 degree, respectively. Similarly, different sized supercells 1 × 1 × 1, 2 × 2 × 2, 3 × 3 × 2, 4 × 4 × 2 were used for the doped matrix. In the case of Ti:GaN, the calculated values of formation energy and total energy (per formula unit of GaN) for 2 × 2 × 2 supercell (32 atoms) increased by 0.10 eV and 0.52 eV, respectively, when compared with that of 4 × 4 × 2 supercell (128 atoms). Taking these values and increase in computational time per N^2^ lnN for N number of atoms, we used 2 × 2 × 2 supercells (with doping concentration 6.25%) for the majority of the calculations. The data presented in the coming sections will be related to the calculations made on 2 × 2 × 2 supercells unless otherwise specified. The doping concentration of single dopants in 32 atom supercell was 6.25% whereas its value for Ti-Ce codoped case was 12.5%. 

The value of heat of formation for pure GaN at 0 K was calculated using $\Delta H_{f}\left[GaN\right]=\mu_{GaN}-\mu_{Ga}-\mu_{N}$ where $\mu_{GaN}=E_{GaN}^{DFT}$. is the calculated value of total energy of bulk GaN and $\mu_{Ga}=E_{Ga}^{DFT}$, $\mu_{N}=E_{N}^{DFT}$ are calculated values per atom for Ga and N, respectively, in their reference phase. We used gallium metal with an orthorhombic structure [165979-ICSD] and solid nitrogen having a tetragonal structure [24892-ICSD] as reference phases in the calculations [14]. 

The anionic chemical potential $\mu_{N}$ is a variable that points to the growth of GaN under N-rich or N-poor or an intermediate condition. The upper and lower limits of $\mu_{N}$ are found by considering GaN in equilibrium nitrogen and gallium reservoirs in bulk. The stable condition for the growth of GaN refers to a value found by summing of $\mu_{N}$ and $\mu_{Ga}$ to ensure $\underset{n}{\sum }(\mu_{Ga,n}+\mu_{N,n})=E\left(Ga_{n}N_{n}\right)$ in equilibrium for n number of atoms in pure GaN. The calculated values however revealed expectedly the availability of excess atomic chemical potentials as $\mu_{Ga}\left(bulk\right)+\mu_{N}\left(bulk\right)$ is found greater than $E\left(Ga_{n}N_{n}\right)$. It, therefore, points to the need of considering extreme limits of cationic and anionic rich conditions to carefully evaluate the heat of formation of the material. In order to consider experimental growth conditions, we assumed Ga-rich and N-rich environments for finding the energy of formation of GaN [15]. For Ga-rich (nitrogen deficient) case chemical potentials of Ga and N were found using $\mu_{Ga}^{Ga-rich}=\mu_{Ga}$ and $\mu_{N}^{Ga-rich}=\mu_{GaN}-\mu_{Ga}$, respectively, to give respective values of −2.90 eV and −9.26 eV. On the other hand, for N-rich conditions, the values of chemical potentials of Ga and N were taken as $\mu_{Ga}^{N-rich}=\mu_{GaN}-\mu_{N}$ and $\mu_{N}^{N-rich}=\mu_{N}$, respectively, to give respective values of −3.95 eV and −8.73 eV. 

The value of formation energy for defect X_Ga_ (where X = Ti, Ce) was calculated using defect reaction $E_{F}=E_{X:GaN}-E_{GaN,bulk}+n.\mu_{Ga\;}–n.\mu_{X}$ + $q.E_{F}$ + where $E_{X:GaN}$ is the calculated value of total energy for doped GaN, $\mu_{Ga}$ and $\mu_{X}$ are respective values of Ga and dopant chemical potential for n number of atoms with respect to the bulk reference, q is the charge state of the dopant in case of charged defects and E_F_ is the energy of the reservoir of electrons. In order to ensure exact stoichiometric conditions, we used averaged values of chemical potential as $\mu_{Ga}=-3.43\;\mathrm{eV}$ and $\mu_{N}=-8.99\;\mathrm{eV}$. These values, subject to pure GaN, will be changed for doped cases. However, this effect may be ignored because doping of 1 × 10^19^ cm^−3^ foreign atoms in wurtzite GaN (8.9 × 10^22^ atoms cm^−3^) will replace one dopant in 8900 host Ga atoms. Metallic Ti with orthorhombic structure [165979-ICSD] and metallic Ce having the tetragonal structure [24892-ICSD] were taken as metallic reference phases for the dopants in the calculations [14]. The fully converged values for bulk Ti and Ce were found to be $\mu_{Ti}=-8.22\;\mathrm{eV}$ and $\mu_{Ce}=-5.91\;\mathrm{eV}$ in metallic reference. On the other hand, TiN in cubic structure [604220-ICSD] and CeN in cubic structure [621558-ICSD] were taken as metallic nitride reference phases. For finding the value of $\mu_{Ti}$ while taking TiN as a reference, we assumed extreme cases of Ti-rich and N-rich situations. In the case of Ti-rich and N-rich environments, the respective values of chemical potentials of Ti were calculated using $\mu_{Ti}^{Ti-rich}=\mu_{Ti,\;metal}$ and $\mu_{Ti}^{N-rich}=\mu_{TiN}-\mu_{N,bulk}$. The average value of $\mu_{Ti}$ for full stoichiometric conditions of reference TiN came out to be −10.67 eV. On the same lines, the calculated value of $\mu_{Ce}$ for full stoichiometric conditions of reference CeN came out to be −11.57 eV.

For each $E_{X:GaN}$ the corresponding $E_{GaN,bulk}$ value was calculated from the same size of supercell. In the case of calculations for the charged state of the dopants X^N+^ (X = Ti, Ce and N = 2, 3, 4) we added the term containing one electron energy (energy of a hole at VBM) by using $E_{N}-E_{N-1}=E_{V}$ to ensure the conservation of mass and charge [16]. The value of energy as reference for electrons was used as $E_{F}=E_{V}+\Delta E_{F}$ where $\Delta E_{F}$ is the position of the Fermi level with respect to VBM which is the energy of the highest occupied level [17,18]. For the realization of dopant charge states X^N+^ we added or removed the suitable number of electrons from supercell containing neutral defect X_Ga_ before calculating total energy. The change in the number of electrons will modify the valance state related to defect X_Ga_ because the highest occupied state corresponds to TM and RE dopants [19].

## 3. Results and Discussion 

### 3.1. Pure Gallium Nitride

The calculated value of heat of formation for pure GaN came out to be −1.055 eV which indicates that the thermodynamically permissible range varies from −1.055 eV (for Ga-rich) to 0 eV (for N-rich). This value is in agreement with reported values calculated with GGA and is close to the experimental value [20,21]. Furthermore, the calculated values of lattice parameters a = b = 3.247 Å, c = 5.281 Å and bandgap 1.62 eV also agree with reported literature [21]. The values of Ga-N bond length in a (or b) and c-axis are 1.983 Å and 1.991 Å, respectively. Our calculated values of c/a (1.626) and u (0.375) deviate from their respective ideal values (1.633, 0.375) by 0.42% and 0%. The variations in values of c/a ratio and u parameter are of interest in characterizing the doping-dependent structural modifications because these parameters act as a probe to explore the deviation of hexagonal structure from the ideal one. The value of structural parameters for Ti-doped GaN and Ce-doped GaN were calculated and discussed in the following sections. 

### 3.2. Ti-Doped GaN

Prior to placing the dopant at a predefined location the natural residing tendency of a Ti atom in 2 × 2 × 2 GaN supercell containing a Ga vacancy was studied. A Ti atom placed at 20% off-cationic site along $\left[11\bar{2\;}1\right]$ in the host matrix was allowed to relax to minimum energy configuration. After running five cycles of geometrical optimization, the Ti atom was settled at a cationic site which indicates Ti_Ga_ as the preferred location of the dopant in GaN. The Ti-N-Ti angle (dihedral angle) is increased by 0.63% when compared with that of Ga-N-Ga angle for pure GaN. The comparison of bond lengths indicates an increase in apical bond length and basal bond length by 0.57% and 1.25%, respectively. In order to further check the lattice site location of the dopant, Ti and neighboring Ga both were oppositely placed 40% off-cationic site along c-axis. 

The computed values of the bond lengths and dihedral angles in the case of pure GaN and Ti:GaN before and after relaxation are given in Table 1.

**Figure 1:**
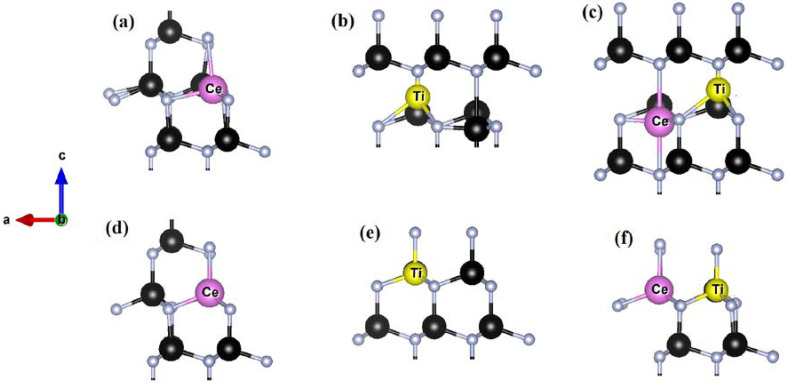
Local geometry of doped GaN (**a**) Ce:GaN with Ce, neighboring Ga, and connecting N atoms displaced along $\left[11\bar{2\;}1\right]$ (**b**) Ti:GaN with Ti and neighboring Ga displaced along along $\left[000\bar{1\;}\right]$ and [0001,] respectively, (**c**) Ti-Ce:GaN with Ce and Ti displaced along $\left[000\bar{1\;}\right]$ and [0001], respectively, (**d**–**f**) shows relaxed structures after geometrical optimization for Ce:GaN, Ti:GaN and Ti-Ce:GaN, respectively. Black and gray spheres represent Ga and N atoms, respectively, whereas the dopant atoms Ti and Ce are already mentioned. Direction indicator showing a-, b- and c-axis is given.

**Figure 2:**
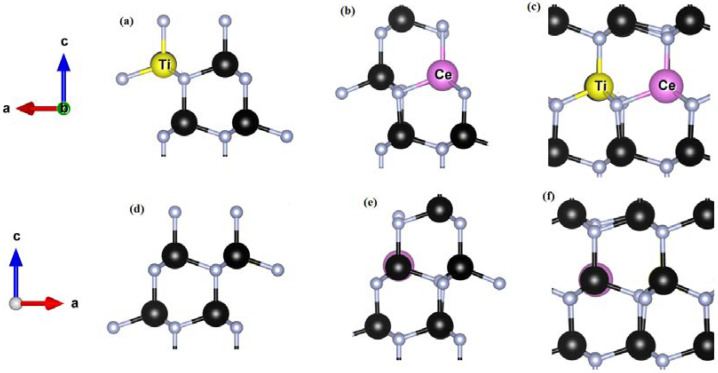
Local geometry of (**a**) Ti_Ga_ (**b**) Ce_Ga_ (**c**) Ti_Ga_-Ce_Ga_ in GaN after complete relaxation of respective structures. (**d**–**f**) shows 180° rotated structures to view the structures from the backside. Black and gray spheres represent Ga and N atoms, respectively, whereas the dopant atoms Ti and Ce are already mentioned. Direction indicator showing a-, b- and c-axis is given.

For the situation in which Ti was shifted towards [0001] and Ga to $\left[000\bar{1\;}\right]$ (Figure 1b) upon full relaxation Ti settled at the cationic site (Figure 1e) with similar bond lengths and angles calculated for the previous case in which Ti was displaced along $\left[11\bar{2\;}1\right]$. Similar results were obtained upon relaxation when Ti and neighboring Ga were shifted 40% off-cationic site towards $\left[000\bar{1\;}\right]$ and [0001], respectively. These tests indicate preferential accommodation of Ti on cationic Ga sites in GaN [22]. Finding it safe and justified, we used Ti_Ga_ for different configurations of Ti:GaN in this work. In the case of Ti_Ga_ it is found that Ti relaxed in such a way that basal and apical Ti-N bond lengths increased by 0.56% and 1.2%, respectively (Figure 2a,d). Furthermore, a widening of dihedral angle by 0.6% is also observed in comparison with pure GaN geometry. 

The analysis of these findings indicates that Ti_Ga_ site is located 0.6% (0.75% per 3 × 3 × 2 supercell calculations) shifted along $\left[000\bar{1\;}\right]$ when compared with regular cationic sites of the matrix (Figure 1a,d). The doping of Ti in the host is also investigated using Hubbard correction with different values of Hubbard parameter U from U = 0 eV to U = 8 eV with a step size of 0.5 eV (Table 2). The value of magnetic moment per Ti is found ~0.683 µ_B_ for U = 0 eV which is found consistently increasing to value 1.306 µ_B_ for U = 8 eV [23]. However, the value 0.972 µ_B_ is assigned as favorable since it is found against U = 4.5 eV that has been reported as a suitable value of the Hubbard parameter for Ti-doped GaN [24]. The in-plane cation–cation nearest neighbor distance which is 6.494 Å for GaN appeared as 6.517 Å for Ti:GaN for U = 0 eV and was found to be consistently increasing for all values of U. However, the values determined against U = 4.5 eV are found 6.524 Å and 10.595 Å along in-plane and out-of-plane axis, respectively. The values of cation-N_i_ (where i represents different atoms) distance are found larger for the doped material and appeared consistently increasing with an increase in value of U [25]. Similarly, the values of Ti-Ga_i_ distance are found larger for the doped material and appeared consistently increasing with an increase in the value of U.

The cationic substitution of Ti as $Ti_{Ga}^{0}+e$, $Ti_{Ga}^{0}$ and $Ti_{Ga}^{0}-e$ produces dopant’s charge states +2, +3, and +4, respectively, in GaN matrix. The calculated values of defect formation energies turned out to be in order Ti^2+^ > Ti^3+^ > Ti^4+^. The formation energy for $Ti_{Ga}^{+2}$ is found sufficiently high to exclude the probability of Ti in +2 whereas the least value of formation energy found in the case of $Ti_{Ga}^{+4}$ predicts the preferential likelihood of Ti in +4 charge state when doped in hexagonal GaN. There is a possibility of the existence of multiple charge states of dopants in semiconductor hosts [26]. In order to check this possibility, Ti dimmer was doped in a mixed charge state of Ti^3+^ and Ti^4+^ (in equal concentration) in GaN. The results indicated that formation energy calculated for the mixed charged state is less than all cases of the single charge state of Ti in GaN [27]. 

The values of formation energy and structural parameters calculated for pure GaN and Ti substituted GaN with dopant charge states Ti^2+^, Ti^3+^ and Ti^4+^ are given in Table 3. The values of in-plane and out-of-plane Ti-N bond lengths, lattice constant ‘a’ and c/a ratio are found to decrease in order Ti^2+^ > Ti^3+^ > Ti^4+^. This trend is expected due to a decrease in atomic radii of the dopant with an increase in its positive charge state [28]. The value of formation energy computed for mixed case Ti^3+^+ Ti^4+^:GaN (2 × 2 × 2) in FM configuration is lower (i.e., −8.233 eV) in comparison to the AFM configuration (i.e., −8.097 eV) which points to settling of Ti^3+^and Ti^4+^ in FM ground state in GaN matrix [29].

The computed value of lattice constant for GaN is 3.247 Å which is increased to 3.287 Å, 3.259 Å, and 3.232 Å for Ti dopant in charge states +2, +3, and +4, respectively [30]. The value of lattice constant in case of charge state +4 (energetically more favorable single Ti dopant in GaN) was also calculated for bigger supercell 3 × 3 × 2 which yielded a value of 3.240 Å that is close to that of pure GaN. The value of Ga-N in-plane bond length is 1.983 Å which changed to 2.008 Å, 1.987 Å, and 1.939 Å for the doped case with dopant charge states +2, +3, and +4, respectively. Whereas, out-of-plane values of Ti-N are found as 2.025 Å, 1.988 Å, and 1.937 Å for the dopant charge states +2, +3, and +4, respectively [31,32]. In the case of the most favorable dopant charge state Ti^4+^ the in-plane and out-of-plane Ti-N bond length values computed in the supercell 3 × 3 × 2 are 1.940 Å and 1.927 Å, respectively. The computed values of the bond lengths, c/a ratio, dihedral angles, internal parameter u, and formation energy for pure GaN and Ti:GaN with dopant charge states +2, +3 and +4 are given in Table 3.

### 3.3. Ce Doped GaN

In order to check the seating preference of the dopant in the case of Ce:GaN, Ce, neighboring N, as well as Ga atoms, were displaced to distort the tetrahedron (Figure 1a). After complete relaxation, Ce and Ga atoms settled at regular cationic sites whereas connecting N atom was found displaced outward in order to accommodate the heavier dopant in the structure (Figure 1d). An increase of 11.1% in basal and 13.5% in apical bond length Ce-N was observed. The dihedral angle should have increased keeping Ce atomic size and outward relaxation of terminating N atoms placed at vertices in tetrahedron [31]. However, a decrease in this angle is observed which quantitatively points to a 2.7% shift of the Ce_Ga_ site along [0001] contrary to that of the Ti_Ga_ case in the host matrix.

The value of basal bond length Ga-N is 1.983 Å which is increased to 1.995 Å when Ti is cationically substituted or when Ti and neighboring Ga both are displaced away prior to the relaxation. On the other hand, in the case of Ce:GaN, Ce relaxed to site with Ce-N bond length 2.204 Å. Similarly, the values of apical bond length for Ti:GaN and Ce:GaN are found to be 2.017 Å and 2.261 Å. It appears that both the dopants relaxed to nearly the same location irrespective of initially placing at the cationic site or off-cationic site. 

The doping of Ce in the host is also investigated using Hubbard correction of U = 6.2 eV [22]. The value of magnetic moment per Ce is found 0.533 µ_B_ for U = 0 eV which is found 0.921 µ_B_ for U = 6.2 eV (Table 4). The in-plane cation–cation nearest neighbor distance which is 6.494 Å for GaN appeared as 6.543 Å for Ce:GaN for GGA and was found to be 6.545 Å when Hubbard correction is switched on consistently increasing for all values of U. The values of cation-N_i_ (where i represents different atoms) distance are found larger for the doped material and appeared further increased in case of GGA + U. Similarly, the values of Ce-Ga_i_ distance are found larger for the doped material and appeared further higher when the correction is applied. In addition to doping of neutral Ce, the cases of charged state dopant in GaN are also investigated. 

### 3.4. Ti-Ce Codoped GaN

The codoping of Ti and Ce in GaN was also studied in detail at GGA and GGA + U levels of theory. The detailed analysis pointed out the cationic substation of individual Ti and Ce atoms upon full relaxation in the GaN matrix [33]. However, keeping in view the exchange interactions of magnetically active atoms Ti and Ce it is better to check the seating priority of these atoms when placed in GaN. An initial structure was prepared in which Ce and Ti were displaced 40% off-cationic sites in opposite directions along the c-axis. Upon full relaxation, Ce settled at site 7.8% away along $\left[000\bar{1\;}\right]$ and Ti adjusted itself at site 14% away along [0001] when compared with regular cationic sites of GaN as per Figure 1c,f. This trend is contrary to that which was observed in individual doped cases of both these atoms. The Ti-Ce distance was not the same when the dopants were placed at cationic sites in comparison to the case when the dopants were displaced away from the lattice sites in opposite directions prior to relaxation [34]. Further, in both these cases, the optimized values of Ti-N, Ce-N, and dihedral angles are also different. The difference in such values is not significant but still not negligible which indicates that structure may have not been trapped into a local minimum but fully relaxed per computational details. It points to the fact that optimized geometry is sensitive to the starting position of the dopants. The computed structural parameters of Ti-Ce codoped GaN before and after geometrical optimization are given in Table 5.

The values of magnetic moment per Ti and Ce atoms in FM configuration appeared as 0.690 µ_B_ and 0.589 µ_B_, respectively, which exhibited a total magnetic moment of 1.356 µ_B_ (Table 6). On the other hand, in the case of AFM configuration, values of magnetic moment per Ti and Ce atoms appeared as 0.691 µ_B_ and −0.589 µ_B_, respectively, which provided a total magnetic moment per supercell as 0.101 µ_B_ [35]. The AFM configuration appeared slightly lower in energy which points to relaxation of the Ti and Ce magnetic moments in the antiferromagnetic coupled ground state in GaN. The distance Ce-N_2_ which was noted at 2.190 Å in the case of Ce:GaN increased to 2.220 Å in the case of Ti-Ce:GaN. On the other hand, the distance Ti-N_4_ which was 1.995 Å is decreased to 1.979 Å in the case of Ti-Ce:GaN. It points to the relaxation of Ce outwards whereas that of Ti inwards with reference to N atoms. The in-plane Ti-Ti distance which was 6.518 Å in the case of Ti:GaN increased to 6.588 Å for Ti-Ce:GaN whereas the Ce-Ce distance which was 6.554 Å in the case of Ce:GaN appeared to be 6.582 Å for the codoped case. The Ga-Ga distance (pure GaN) of 3.247 Å is increased to the Ti-Ce separation of 3.294 Å which also points to the outwards relaxation of the dopants. Due to atomic size differences, the separation Ti-Ce (Figure 2c) may give only a quantitative comparison with Ga-Ga separation. However, the dihedral angle Ti-N-Ce may give a qualitative picture to shed light on Ti-Ce interactions compared with the angle Ga-N-Ga. The computed results reveal a notable decrease in angle from 109.84° (Ga-N-Ga for pure GaN) to 102.10° (Ti-N-Ce for pure GaN). In the case of Ti:GaN the angle Ti-N-Ga was 109.68° whereas the value of angle Ce-N-Ga was 103.51° which indicates that these values are less than that of pure GaN but higher than that of the codoped case. The magnetic exchange interactions between Ti and Ce ions are responsible for the preferential decrease in Ti-N-Ce angle [36]. The lowest value of angle Ti-N-Ce points to the nearness of Ti and Ce ions that may have taken place due to magnetic exchange interactions between them.

Experimental doping and actual manufacturing of the materials to add foreign atoms during growth is carried out for different applications [37]. This work is carried out with the motivation to shed light on the dopant location mechanism during experimental doping by performing theoretical calculations. The starting position of the dopant will be changed when structural relaxation is carried out during geometry optimization. The same is true in the introduction of the dopants either during in situ doping or via ion-implantation [38]. Thermal annealing of the doped/implanted samples helps to settle the dopant atoms at minimum energy sites in the host for their electrical, optical, and magnetic activation.

## 4. Conclusions

In order to check the lattice site location of the individual dopants Ti, Ce and codoped Ti + Ce in wurtzite GaN, (i) the dopants were placed at cationic sites (ii) the dopants and neighboring Ga both were oppositely placed 40% off-cationic site along c-axis. The geometry optimization relaxed Ti on cationic Ga sites when individually doped in GaN. The detailed analysis pointed out the cationic substitution of individual Ti and Ce atoms upon full relaxation in the GaN matrix. However, in the case of Ti-Ce codoping, Ce settled at site 7.8% away along $\left[000\bar{1\;}\right]$ and Ti adjusted itself at site 14% away along [0001] from regular cationic sites. This trend is contrary to that what was observed in individual doped cases of both these atoms. The analysis of the results indicates that optimized geometry is sensitive to the starting position of the dopants.

## Figures and Tables

**Table 1:** Calculated structural parameters before and after relaxation for pure GaN, Ti substituted GaN, Ti, Ga placed off-cationic sites in Ti:GaN.

Configuration	Before Relaxation				After Relaxation			
	Ti-N Basal Bond Length (Å)	Ti-N Apical Bond Length (Å)	Ti-N-Ga Angle (Degree)	N-Ti-N Angle (Degree)	Ti-N Basal Bond Length (Å)	Ti-N Apical Bond Length (Å)	Ti-N-Ga Angle (Degree)	N-Ti-N Angle (Degree)
Pure GaN	1.654	1.652	109.36	109.36	1.983	1.991	109.84	109.84
Ti substituted GaN	1.670	1.706	108.92	112.40	1.995	2.017	109.68	110.54
Ti displaced up Ga displaced down	2.306	1.205	115.78	87.48	1.995	2.016	109.68	110.55
Ti displaced down Ga displaced up	1.845	2.706	115.78	119.63	1.995	2.017	109.68	110.56
Ti displaced along a, b, c	1.924 2.089 2.167	1.883	111.38	107.14 101.44 110.30	1.995	2.016	109.71	110.54

**Table 2:** The values of magnetic moment, and structural parameters computed for pure GaN and Ti:GaN without and with Hubbard correction. The values of Ti-Ti nearest neighbor distance along three axes and distance of Ti to different N atoms and G atoms are also given.

Material/Details	Magnetic Moment (µ_B_)		Ti-Ti NN Distance along All Axis			Ti-N Interatomic Distance (Å)				Ti-Ga Interatomic Distance (Å)					
	Per Ti	Total	a	b	c	Ti-N_4_	Ti-N_6_	Ti-N_8_	Ti-N_16_	Ti-Ga_3_	Ti-Ga_5_	Ti-Ga_6_	Ti-Ga_7_	Ti-Ga_8_	Ti-Ga_15_
Pure GaN	0	--	6.494	6.494	10.562	1.983	1.983	1.983	1.991	3.238	3.238	3.238	3.238	3.238	5.281
Ti-doped (displ.)	0.683	0.709	6.518	6.518	10.583	1.995	1.995	1.995	2.016	3.280	3.279	3.242	3.279	3.241	5.322
Ti-doped (U = 0)	0.684	0.710	6.517	6.517	10.584	1.995	1.995	1.995	2.017	3.280	3.280	3.241	3.280	3.241	5.324
Ti-doped (U = +1.0)	0.714	0.736	6.518	6.518	10.585	2.000	2.000	2.000	2.024	3.282	3.282	3.242	3.282	3.242	5.325
Ti-doped (U = +1.5)	0.741	0.750	6.517	6.517	10.584	1.995	1.995	1.995	2.017	3.280	3.280	3.241	3.280	3.241	5.324
Ti-doped (U = +2.0)	0.774	0.786	6.520	6.520	10.587	2.010	2.010	2.010	2.039	3.285	3.285	3.244	3.285	3.244	5.327
Ti-doped (U = +2.5)	0.804	0.811	6.521	6.521	10.589	2.015	2.015	2.015	2.047	3.287	3.287	3.246	3.287	3.246	5.329
Ti-doped (U = +3.0)	0.833	0.835	6.522	6.522	10.590	2.021	2.021	2.021	2.056	3.290	3.290	3.247	3.290	3.247	5.331
Ti-doped (U = +3.5)	0.861	0.858	6.523	6.523	10.592	2.026	2.026	2.026	2.064	3.292	3.292	3.248	3.292	3.248	5.334
Ti-doped (U = +4.0)	0.876	0.872	6.524	6.524	10.595	2.028	2.028	2.028	2.076	3.300	3.300	3.245	3.300	3.245	5.342
Ti-doped (U = +4.5)	0.972	0.912	6.524	6.524	10.595	2.040	2.040	2.040	2.092	3.298	3.298	3.249	3.298	3.249	5.338
Ti-doped (U = +5.0)	0.946	0.882	6.526	6.526	10.601	2.038	2.038	2.038	2.105	3.311	3.311	3.243	3.311	3.243	5.244
Ti-doped (U = +5.5)	0.986	0.895	6.527	6.527	10.605	2.047	2.047	2.047	2.105	3.305	3.305	3.256	3.305	3.256	5.261
Ti-doped (U = +6.0)	1.032	0.911	6.527	6.527	10.610	2.055	2.055	2.055	2.110	3.302	3.302	3.266	3.302	3.266	5.272
Ti-doped (U = +6.5)	1.093	0.933	6.528	6.528	10.615	2.063	2.063	2.063	2.117	3.301	3.301	3.274	3.301	3.274	5.281
Ti-doped (U = +7.0)	1.187	0.969	6.529	6.529	10.620	2.076	2.076	2.076	2.132	3.301	3.301	3.279	3.301	3.279	5.288
Ti-doped (U = +7.5)	1.246	0.990	6.529	6.529	10.625	2.084	2.084	2.084	2.141	3.301	3.301	3.286	3.301	3.286	5.296
Ti-doped (U = +8.0)	1.306	1.009	6.530	6.530	10.631	2.092	2.092	2.092	2.151	3.301	3.301	3.293	3.301	3.293	5.304

**Table 3:** The values of structural parameters and formation energy calculated for pure GaN, Ti:GaN with dopant charge states Ti^2+^, Ti^3+^, and Ti^4+^.

Material	Lattice Constants a = b (Å)	c/a Ratio	Ga-N (for GaN) Ti-N (for Ti:GaN) Bond Length (Å)		Angle (Degree)			Internal Parameter u (Å)	Formation Enthalpy/Defect Formation Energy (eV)
			In-Plane	Out-of-Plane	Ga-N-Ga	N-Ti-N	Ti-N-Ga		
GaN (2 × 2 × 2)	3.247	1.6263	1.983	1.991	109.84	109.84	109.84	0.375	−1.055
Ti^2+^:GaN (2 × 2 × 2)	3.287	1.6284	2.008	2.025	109.89	110.79	109.58	0.375 0.377	5.325
Ti^3+^:GaN (2 × 2 × 2)	3.259	1.6238	1.987	1.988	110.00	110.54	109.68	0.375 0.377	−0.065
Ti^4+^:GaN (3 × 3 × 2)	3.240	1.6240	1.939	1.937	109.90	110.46	111.10	0.382 0.368	−0.598
Ti^4+^:GaN (2 × 2 × 2)	3.232	1.6190	1.940	1.927	108.85	110.50	110.50	0.380 0.370	−0.432

**Table 4:** The values of magnetic moment, and structural parameters computed for pure GaN and Ce:GaN without and with Hubbard correction.

Material/Details	Magnetic Moment (µ_B_)		Ce-Ce (nn Distance) along All Axis (Å)			Ce-N Interatomic Distance (Å)				Ce-Ga Interatomic Distance (Å)					
	Per Ce	Total	a	b	c	Ce-N_2_	Ce-N_4_	Ce-N_8_	Ce-N_12_	Ce-Ga_2_	Ce-Ga_3_	Ce-Ga_4_	Ce-Ga_7_	Ce-Ga_8_	Ce-Ga_15_
Pure GaN	0	--	6.494	6.494	10.562	1.983	1.983	1.983	1.991	3.238	3.238	3.238	3.238	3.238	3.247
Ce doped (displaced)	0.533	0.595	6.554	6.567	10.606	2.190	2.203	2.198	2.272	3.296	3.307	3.297	3.372	3.345	3.335
Ce doped MAG	0.527	0.589	6.543	6.543	10.658	2.205	2.205	2.205	2.261	3.392	3.309	3.392	3.309	3.392	3.272
Ce doped (U = +6.2)	0.921	0.923	6.545	6.545	10.672	2.246	2.246	2.246	2.316	3.423	3.300	3.423	3.300	3.423	3.275

**Table 5:** Structural parameters of Ti-Ce codoped GaN before and after geometrical optimization.

Configuration	Before Relaxation								After Relaxation							
	Basal Bond Length (Å)		Apical Bond Length (Å)		Ti-Ce Distance(Å)	Angle (Degree)			Basal Bond Length (Å)		Apical Bond Length (Å)		Ti-Ce Distance(Å)	Angle (Degree)		
	Ti-N	Ce-N	Ti-N	Ce-N		N-Ce-N	N- Ti-N	Ce-N-Ti	Ti-N	Ce-N	Ti-N	Ce-N		N-Ce-N	N-Ti-N	Ce-N-Ti
Pure GaN	1.654	1.654	1.652	1.652	2.699	109.36	109.36	109.36	1.983	1.983	1.991	1.991	3.247	109.84	109.8	109.84
Ce-Ti:GaN	1.991	1.995	1.992	2.017	3.259	110.54	108.81	109.68	1.985	2.227	2.037	2.294	3.294	110.71	94.43	102.60
Ce(up)-Ti(down): GaN	2.306	1.845	3.410	4.183	3.525	119.63	108.48	115.78	1.984	2.227	2.037	2.293	3.290	110.69	94.40	102.59

**Table 6:** The values of magnetic moment, and structural parameters computed for pure GaN, and Ti-Ce:GaN without and with Hubbard correction for FM and AFM configurations. The values of Ce-Ce and Ti-Ti nearest neighbor separation in a, b and c-axis are given. The dopant sites in the host were switched as per given data.

Material/Details	Magnetic Moment (µ_B_)			Ce-Ce (nn Distance) along All Axis (Å) Ti-Ti (nn Distance) along All Axis (Å)			Ti-Ce (Å)	Ce-N Interatomic Distance Ti-N Interatomic Distance (Å)				Ce-Ga Interatomic Distance (Å) Ti-Ga Interatomic Distance (Å)			Ti-N-Ce Angle (°)
	Per Ce	Per Ti	Total	a	b	c		Ce-N_2_Ti-N_4_	Ce-N_4_Ti-N_6_	Ce-N_8_Ti-N_8_	Ce-N_12_Ti-N_16_	Ce-Ga_11_Ti-Ga_14_	Ce-Ga_14_Ti-Ga_11_	Ce-Ga_10_Ti-Ga_13_	
Pure GaN	--	--	0	6.494	6.494	10.562	3.247	1.983	1.983	1.983	1.991	5.281	6.199	3.247	109.84
Ti-Ce:GaN (GGA)	0.168	−0.049	0.103	6.582 6.588	6.588 6.588	10.727 10.727	3.294	2.220 1.979	2.225 1.979	2.225 2.006	2.299 1.997	5.364 5.320	6.296 6.327	3.270 3.350	102.10
Ti-Ce:GaN (GGA) FM (Ce_Ga16_ and Ti_Ga12_)	0.690	0.589	1.356	6.580 6.580	6.591 6.591	10.729 10.729	3.291	2.228 1.998	2.228 1.985	2.228 1.985	2.294 2.037	5.380 5.372	6.297 6.299	3.259 3.321	102.61
Ti-Ce:GaN (GGA) AFM (Ce_Ga16_ and Ti_Ga12_)	0.691	−0.589	0.101	6.580 6.580	6.591 6.591	10.729 10.729	3.292	2.228 1.998	2.228 1.985	2.228 1.985	2.294 2.037	5.380 5.372	6.306 5.357	3.333 3.270	102.60
